# Evaluation of GeneXpert EV assay for the rapid diagnosis of enteroviral meningitis: a systematic review and meta-analysis

**DOI:** 10.1186/s12941-022-00517-3

**Published:** 2022-06-09

**Authors:** Min Lin, Yun-Ran Li, Qi-Wen Lan, Li-Jun Long, Jia-Qi Liu, Ying-Wen Chen, Xun-Jie Cao, Ge-Yuan Wu, Ya-Ping Li, Xu-Guang Guo

**Affiliations:** 1 Department of Clinical Laboratory Medicine, The Third Affiliated Hospital of Guangzhou Medical University, Guangzhou, 510150 China; 2 Department of Chinese and Western Medicine in Clinical Medicine, The Clinical School of Chinese and Western Medicine, Guangzhou Medical University, Guangzhou, 511436 China; 3 Department of Medical Imageology, The Second Clinical School of Guangzhou Medical University, Guangzhou, 511436 China; 4 Department of Medical Laboratory Technology, The KingMed School of Laboratory Medicine, Guangzhou Medical University, Guangzhou, 511436 China; 5 Department of Clinical Medicine, The Third Clinical School of Guangzhou Medical University, Guangzhou, 511436 China; 6 Department of Clinical Medicine, The Second Clinical School of Guangzhou Medical University, Guangzhou, 511436 China; 7 Key Laboratory for Major Obstetric Diseases of Guangdong Province, The Third Affiliated Hospital of Guangzhou Medical University, Guangzhou, 510150 China; 8 Department of Laboratory Medicine, Key Laboratory of Reproduction and Genetics of Guangdong Higher Education Institutes, The Third Affiliated Hospital of Guangzhou Medical University, Guangzhou, 510150 China

**Keywords:** GeneXpert EV, Enterovirus meningitis, PCR, Systematic-review, Meta-analysis

## Abstract

**Background:**

GeneXpert enterovirus Assay is a PCR-based assay for Enterovirus meningitis diagnosis. However, there is currently no research about the performance of GeneXpert enterovirus assay in the diagnosis of enterovirus meningitis. Thus, a systematic review and meta-analysis is significant on the topic.

**Methods:**

Embase, Cochrane Library, Web of Science, and PubMed were systematically reviewed with retrieval types. Some criteria were used to filter the studies. Only studies published in English, that made a comparison between GeneXpert enterovirus assay and RT-PCR, and could be formulated in a 2*2 table, were included. The quality of the included studies was evaluated by QUADAS-2. The effect of the GeneXpert enterovirus assay was assessed by the Sensitivity, Specificity, Positive Likelihood Ratio, Negative Likelihood Ratio, Diagnosis Odds Ratio, and summary receiver operating characteristic (SROC) curve. Publication bias and heterogeneity were evaluated by the Deeks' funnel test and Bivariate Box plot respectively.

**Results:**

7 studies were recruited in the analysis. The Pooled Sensitivity was 0.96 [95% CI (0.94–0.97)], Pooled Specificity was 0.99 [95% CI (0.98–0.99)], Positive Likelihood Ratio was 130.46 [95% CI (35.79–475.58)], Negative Likelihood Ratio was 0.04 [95% CI (0.02–0.10)], and Diagnostic Odds Ratio was 3648.23 (95% CI [963.99–13,806.72)]. In SROC Curve, Area Under Curve (AUC) was 0.9980, and Q*= 0.9849. In Deeks' funnel test, the P-value was 0.807 (P > 0.05), indicating no publication bias. The Bivariate Box plot indicated no evident heterogeneity.

**Conclusions:**

The GeneXpert enterovirus assay demonstrated high diagnostic accuracy in diagnosing enterovirus meningitis.

**Supplementary Information:**

The online version contains supplementary material available at 10.1186/s12941-022-00517-3.

## Introduction

The Enterovirus genus is one of the most populous in the family Picornaviridae [1], in which, human Enteroviruses (Human EVs) have been discovered with more than 250 subtypes and with a diameter between 28 and 30 nm [2]. Meningitis contains bacterial meningitis and aseptic meningitis. As one of aseptic meningitis, which is infected by an enterovirus, [3] Enterovirus meningitisis the most common non-bacterial meningitis and accounts for 90% in children and adults. However, no antiviral drugs have been approved for the treatment of EV infections [4]. In addition, most patients with enterovirus meningitis have no obvious symptoms after infection, with only less than 10 percent of patients behave obvious symptoms and can receive treatment timely [2]. If it is hard to quickly and accurately diagnose whether it's enterovirus meningitis, patients might receive unnecessary treatment or hospitalization [5, 6]. Therefore, simple and reliable methods of identifying patients whether infected with enterovirus meningitis are critical to the clinic.

Nucleic acid amplification methods (NAATs) are widely used in the diagnosis of enterovirus meningitis [7]. The real-time polymerase chain reaction (RT-PCR) is a kind of NAATs. In 2003, the World Health Organization (WHO) announced the adoption of RT-PCR to detect enteroviruses meningitis. Compared with the traditional viral culture [8, 9], RT-PCR can make a diagnosis within a shorter time of 7 h, and with higher sensitivity and specificity (approximately 100%) [10, 11]. Nowadays, RT-PCR is considered the gold standard for the diagnosis and identification of enterovirus meningitis [12]. But RT-PCR assay has two evident limitations. On the one hand, it is too expensive and couldn’t be applied in budget-limited rural clinics in some regions. On the other hand, when detecting CSF samples from low viral load level, like the early phase of infection, its sensitivity is too low to detecting enteroviruses meningitis [13, 14].

The GeneXpert EV assay (GXEA, Cepheid, Sunnyvale, CA) is a fully integrated automated nucleic acid sample preparation system that consists of instruments, computers, and disposable fluid boxes [9], with a diagnostic turnaround time of 2.5 h [15]. What’s more, because its test box is completely independent and could conduct all the procedures, there is less FP in GeneXpert EV Assay in the diagnosis of enteroviral meningitis [9, 16]. However, so far, there is no comprehensive and systematic study for GeneXpert EV assay. Thus, in the present work, we conducted a systematic review and meta-analysis to explore the diagnostic accuracy of GeneXpert EV assay in enteroviral meningitis.

## Methods

### Search methods

Original studies published in English from the establishment of the databases to January 30, 2021, were searched on PubMed, Embase, Cochrane Library, and Web of Science by two researchers (ML, YRL) independently. In addition, the references of the included literature and unpublished literature were hand-searched by two researchers (JQL, ML) to make sure all the relevant articles are covered. Discrepancies were resolved by discussion among all the researchers. The MeSH terms and search strategies are reported in (Additional file 1): appendix S1.

### Study selection

All researchers have received prior training in systematic reviews and meta-analysis. The Kappa test was used to assess the agreement among the results given by the researchers. 50 citations are selected randomly to screen and calculate the Kappa value. If Kappa was less than 0.75, the second round of training will be carried out. Titles and abstracts are screened by two researchers independently (YWC, XJC). The screening of full texts will be screened by two other researchers (ML, GYW). Disagreements were resolved by consensus of all researchers. Overall, studies eligible for inclusion met all of the following criteria: (1) Published in English; (2) GeneXpert EV assay as an index text; (3) RT-PCR as the reference standard; (4) Data in studies could be formed a 2*2 table. The criteria for exclusion were as follows: (1) Non-English pieces of literature; (2) The index text was no GeneXpert EV assay; (3) The reference standard was no RT-PCR; (4) The article data was not enough to formulate the 2*2 table; (5) Studies included conference abstracts or reviews were also excluded. The Preferred Reporting Items for Systematic Reviews and Meta-Analyses (PRISM) was used to describe the steps of the study selection in the systematic review and meta-analysis.

### Data extraction

The name of the first author, year of publication, country, experiment type (prospective or retrospective), the source and quantity of samples, patient's age and gender, index test, gold standard, true-positive (TP), false-positive (FP), true-negative (TN) and false-negative (FN) were extracted from each included study. Due to the limitation of datasets, the classification of RT-PCR couldn’t be extracted from the included studies. Data were extracted by 7 researchers of the study team (ML, YRL, GYW, QWL, WHY, YPL, and JQL) independently. They were blinded to each other’s results. Disagreements were resolved by consensus.

### Quality assessment

Quality assessment of each article was conducted independently by 6 researchers (ML, YRL, JQL, YWC, XJC, YPL) according to the Quality Assessment of Studies of Diagnostic Accuracy included in Systematic Reviews-2 (QUADAS-2) [17]. The QUADAS-2 includes 4 parts: indicator testing, reference criteria, patient selection, and process and time. And each section could be considered as high, unclear, or low risk of bias independently. All analyses were done with Excel and Review Manager 5.3.0 (The Cochrane Collaboration, Copenhagen, Denmark). Disagreements will be discussed and decided by the above 6 researchers.

### Statistical analysis

The Meta-Disc [18] was applied to analyze the pooled sensitivity, pooled specificity, diagnostic positive likelihood ratio (+ LR), diagnostic negative LR (-LR), diagnostic odds ratio (DOR), and SROC curve with all the 95% confidence intervals (95% CI). The publication bias of sensitivity and specificity in the included studies was analyzed in Stata12.0 with Deeks’ funnel plot. In addition, heterogeneity was evaluated according to the Bivariate Box plot by using Stata12.0. In the end, the table checklist of this systematic review and meta-analysis could be found in Additional file 2.

## Results

### Eligible studies after systematic review

36 citations were obtained totally, including 33 citations from the systematic review, and 3 citations from hand-searched. According to the inclusion and exclusion items, 7 citations were left [12, 16, 19–22]. In Katja Seme’s work, preliminary results were obtained by using RT-PCR and GeneXpert EV assay detection. However, due to invalid results existing (preliminary negative and uncertain), the authors reexamined and obtained the final results [19]. Thus, the datasets from Katja Seme were divided into two 2*2 tables. The search process and results according to PRISMA 2009 Flow Diagram are outlined in Additional file 3.

### Study characteristics of the included studies

In all included studies, 2 articles were retrospective [21], and 1 article was both retrospective and prospective [16], only 4 articles provided the ages of the patients clearly [12, 16, 20, 21]. All the studies did not use platforms. The characteristics were summarized in Table 1.

**Table 1:** Summary of the characteristics of included studies

First author	Year	Country	Study design	The specimen source	Age of patients	Patients with gender	Initial sample size	Research involved	Test method
Frederick S Nolte [16]	2010	Georgia	Prospective and retrospective	CSF	156neonates 227Children 53Adults	ALL	475	416	GXEA
Katja Seme (a) [19]^*^	2008	The Republic of Slovenia	Prospective	CSF	UC	UC	162	162	GXEA
Katja Seme (b) [19]^$^	2008	The Republic of Slovenia	Prospective	CSF	UC	UC	162	162	GXEA
Marlowe, E. M. [15]	2008	USA	Retrospective	CSF	UC	UC	138	136	GXEA
Ninove, L. [12]	2011	France	Prospective	CSF	< 1 year,1–4 years,5–14 years,15–24 years,25–49 years, > 50 years	ALL	310	310	GXEA
Ninove, Laetitia [12]	2010	France	Prospective	CSF	ALL	UC	469	390	GXEA
S.C.M. de Crom [21]	2011	Netherlands	Retrospective	CSF	0–84.10	ALL	116	232	GXEA
Slika, S. [22]	2012	USA	Prospective	CSF	UC	UC	220	220	GXEA

First author	Positive	Negative	Reference standard	Positive	Negative	TP	FP	TN	FN
Frederick S Nolte [16]	114	312	RT-PCR	94	322	90	14	308	4
Katja Seme (a) [19]^*^	75	87	RT-PCR	83	79	75	0	79	8
Katja Seme (b) [19]^$^	82	80	RT-PCR	83	79	82	0	79	1
Marlowe, E. M. [15]	25	111	RT-PCR	25	111	25	0	111	0
Ninove, L. [12]	85	174	RT-PCR	81	225	81	0	172	2
Ninove, Laetitia [12]	109	289	RT-PCR	108	352	105	2	283	0
S.C.M. de Crom [21]	32	185	RT-PCR	40	192	32	0	178	7
Slika, S. [22]	42	178	RT-PCR	42	178	42	0	178	0

CSF, cerebrospinal fluid; UC, unclear; TP, ture positive; FP, false positive; TN, ture negative; FN, false negative

^*^In this search, preliminary results using RT-PCR and GeneXpert EV assay detection

^$^In this search, final results were obtained by reexamining the preliminary negative and uncertain results

### Quality assessment of the included studies according to QUADAS-2

**Fig. 1:**
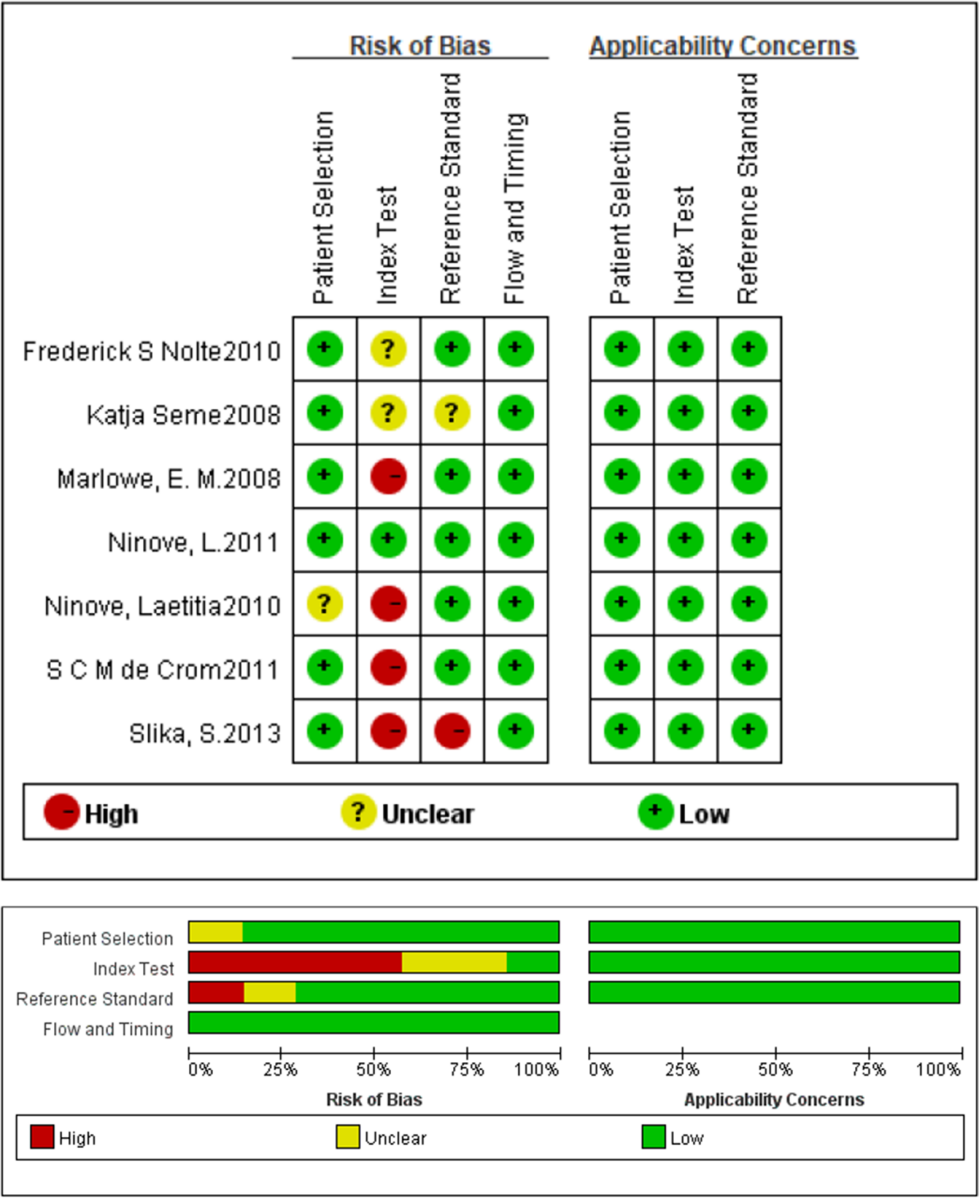
Risk of bias and applicability concerns graph

In Patient Selection, all the studies were considered as low risk of bias. In Index Text, 2 articles were considered as unclear risk of bias because the results of the gold standard and index test were given at the same time; 4 articles were likely to have a high risk of bias because the results of the index test were not carried out without knowing the results of the gold standard. In Reference Standard, 2 studies were likely to have an unclear risk and a high risk of bias, respectively. In Flow and Timing, all studies were likely to have a low risk of bias. The results were plotted in Fig. 1.

### Diagnostic accuracy of expert EV assay for enteroviral meningitis

**Fig. 2:**
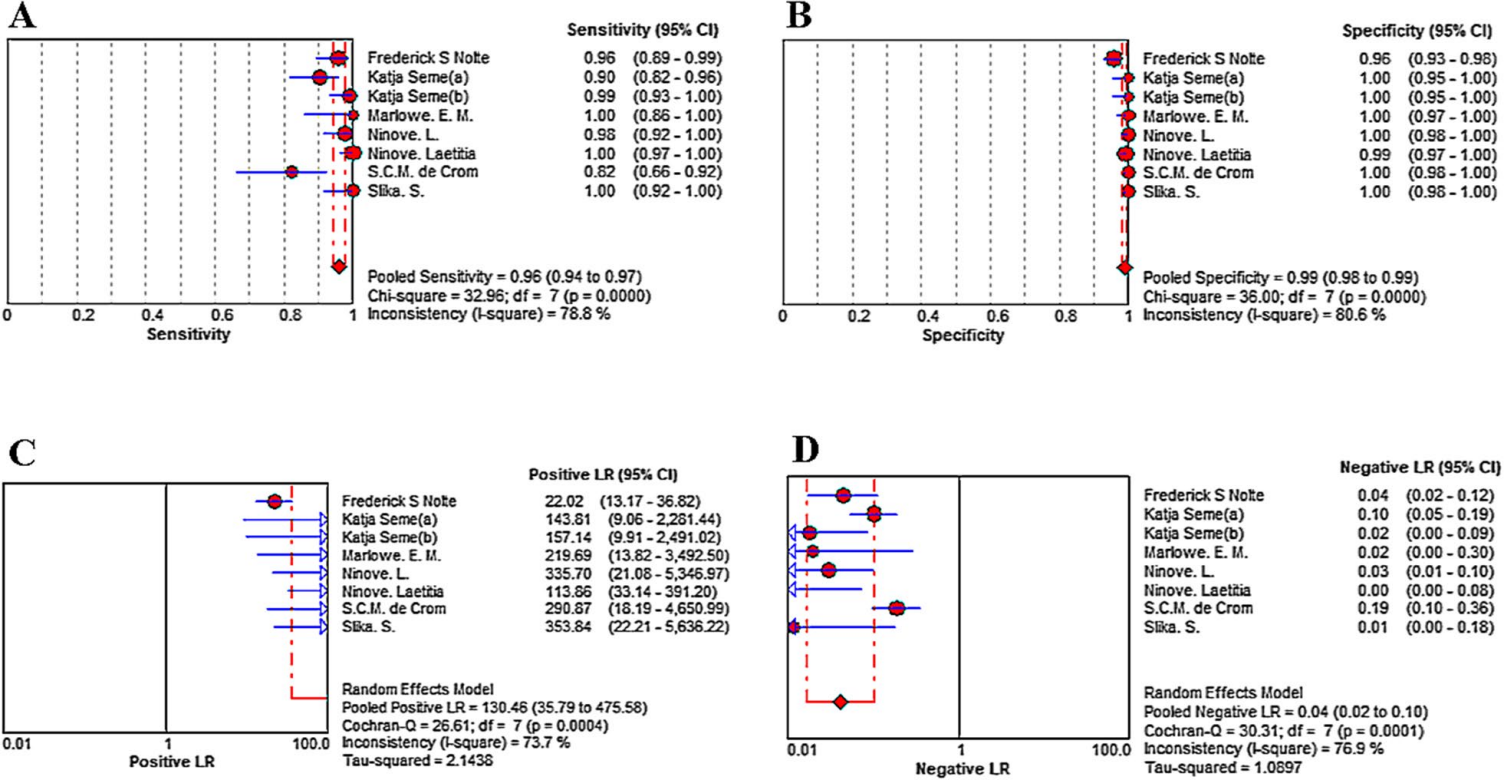
Sensitivity (**A**), specificity (**B**), PLR (**C**) and NLR (**D**)

**Fig. 3:**
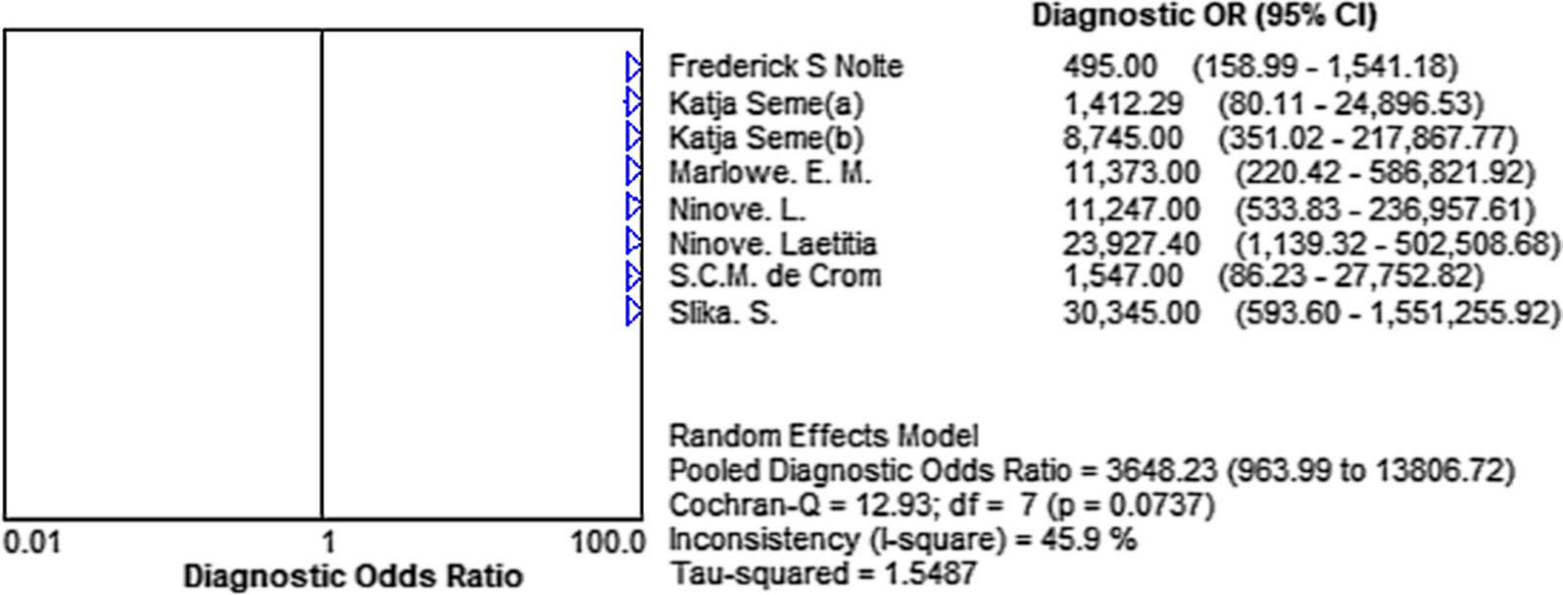
Diagnostic Odds Ratio (DOR)

The Pooled Sensitivity (A) was 0.96 [95% CI (0.94–0.97)], Pooled Specificity(B) was 0.99 [95% CI (0.98 – 0.99)], Pooled Positive LR (C) was 130.46 [95% CI (35.79–475.58)], Pooled Negative LR (D) was 0.04 [95% CI (0.02–0.10)], and Pooled Diagnostic Odds Ratio was 3648.23 (95% CI [963.99–13,806.72)] (Figs. 2A–D, 3.)

### SROC curve of Xpert EV assay for enteroviral meningitis

**Fig. 4:**
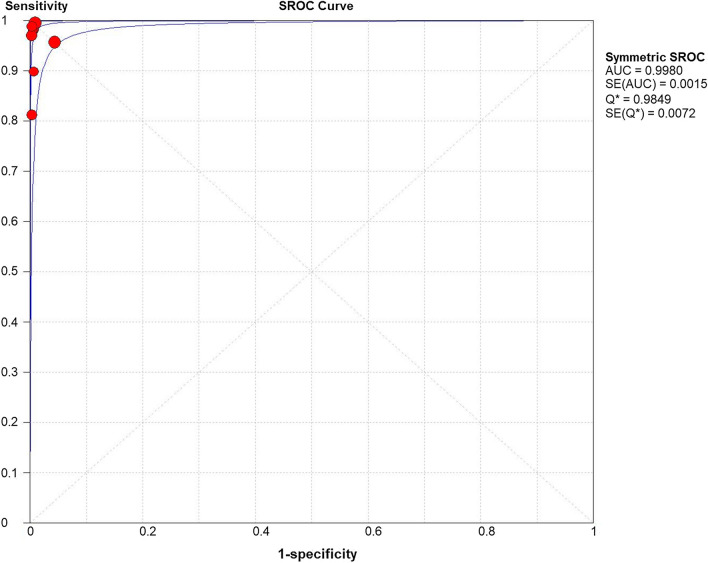
SROC Curve

In SROC Curve (Fig. 4.), AUC was 0.9980, Q index was 0.9849. Both results were close to 1, indicating the high accuracy of the GeneXpert EV in the diagnosis of enteroviral meningitis.

### Publication bias of the included studies

**Fig. 5:**
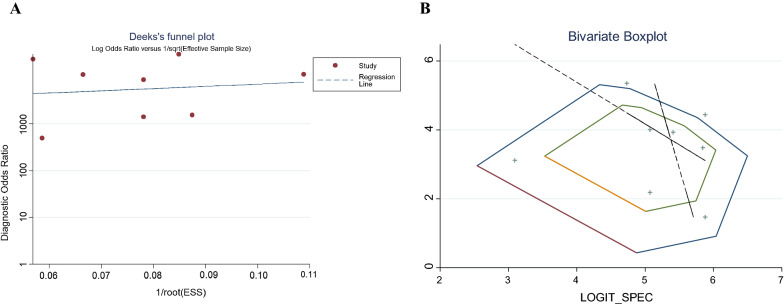
Deeks' funnel plot (**A**) and Bivariate box plot (**B**)

Deeks' funnel plot test was conducted to evaluate the publication bias. In the results, most of the points are symmetrical and the P-value was 0.807 (P > 0.05), indicating that no publication bias exists [23]. The Deeks' funnel plot result could be sought in Fig. 5A.

### Heterogeneity analysis

The Bivariate Box plot shows no evident heterogeneity among the included studies (Fig. 5B). The Pooled Diagnostic Odds Ratio shows no significant heterogeneity through the random-effects model (I^2^ = 45.9% < 50%, Cochran-Q = 12.93, Fig. 3).

## Discussion

In this systematic review and meta-analysis, we evaluated the GeneXpert EV assay in the diagnosis of enterovirus meningitis. On the one hand, the results demonstrate that GeneXpert EV assay has high sensitivity and specificity, although one outlier exists with a lower sensitivity of 0.82 [95% CI (0.66–0.92)]. The outlier may cause by the two-step PT-PCR, which was considered with a higher diagnosis performance than one-step RT-PCR [21]. In Positive LR, a numerical value is lower than others {22.02 [95% CI (13.17–36.82)]}. The lower value can be explained by intricate operations of the equipment with the assumption of FP results may not cause by a target and amplicon cross-contamination [16].

On the other hand, no evident heterogeneity exists in the Bivariate Box plot. In addition, there was no curve pattern (shoulder-arm pattern) in the SROC Curve [18], indicating no evident heterogeneity. Furthermore, the inconsistency was 45.9% in the frost plot of Diagnostic Odds Ratio, which proved the above results once again. However, the inconsistency was 78.8% in the frost plot of Sensitivity, which was prompting the high heterogeneity in the study. Thus, we explored the sources of the heterogeneity: First, the lower sensitivity in the GeneXpert EV assay may be a result of lacking a 1:5 sample dilution of CSF in saline, or freezing and thawing the undiluted CSF [24]. Second, the performance of the GeneXpert EV assay may be influenced by the different lumbar puncture practices in different regions [19]. Unfortunately, since the lack of information, we couldn’t extract this data from included studies.

Undoubtedly, some advantages are represented in the GeneXpert EV assay. First, since the GeneXpert EV assay cartridge is completely self-contained and performs all assay steps including sample preparation, the false positive due to cross-contamination could be avoided. Second, the GeneXpert EV assay is robust and not prone to operator error [16, 19]. Third, reliable results could be obtained rapidly (only 2.5 h) within the spinal fluid collection when using GeneXpert EV assay [25]. Fourth, the GeneXpert EV assay is a fully automatic method, that could be operated easily that specially trained laboratory staff were not required [16].

There are several drawbacks to this systematic review. On the one hand, we only brought English studies into consideration in our study, omitting studies in different languages. On the other hand, there were only 8 sets of data in this analysis due to the insufficiency of qualified studies. Besides, we failed to extract part of the data due to the limitation of data sources.

## Conclusions

In general, the GeneXpert EV assay in diagnosing enteroviral meningitis has been further studied through systematic review and meta-analysis in this work. The results demonstrate that the GeneXpert EV assay has shown good performance in the diagnosis of enterovirus meningitis. As a supplementary method for enterovirus meningitis diagnosis, the GeneXpert EV assay is worthy to be popularized in clinical practice. However, more clinical studies are needed to further explore its role in different viral loads and different patients.

## Supplementary Information

**Additional file 1: S1.** Search terms and search strategy.

**Additional file 2: S2.** Table Checklist of this systematic review and meta-analysis.

**Additional file 3: S3.** Screening processes.

## Data Availability

All data analyzed in this study are included in the article.

## Abbreviations

EV: Enteroviral
QUADAS: Quality assessment of diagnostic accuracy studies
NLR: Negative likelihood ratio
PLR: Positive likelihood ratio
CI: Confidence interval
DOR: Diagnostic odds ratio
SROC: Summary receiver operating characteristic
AUC: Area under the curve
