# Supplementary material for: Evaluation of GeneXpert EV assay for the rapid diagnosis of enteroviral meningitis: a systematic review and meta-analysis
Source: Ann Clin Microbiol Antimicrob. 2022 Jun 9;21:25. doi: 10.1186/s12941-022-00517-3 (PMC9185958; doi:10.1186/s12941-022-00517-3)
Supplement: Supplementary file 1 — Additional file 1: S1. Search terms and search strategy. [file 12941_2022_517_MOESM1_ESM.docx]

**Search terms and strategies of all databases**

**A. Search terms**

Xpert EV; Xpert enterovirus

Meningitis; leptospirotic meningitis; meningeal inflammation; meningitis, recurrent; perimeningeal infections; recurrent meningitis; Meningitides; Pachymeningitis; Pachymeningitides

Enterovirus; Coxsackie virus; coxsackievirus; coxsackieviruses; entero virus; enteroviruses; virus, coxsackie; Coxsackieviruses; Coxsackie Viruses

**B. Search strategies in all databases**

**a. Embase**

('meningitis' OR 'leptospirotic meningitis' OR 'meningeal inflammation' OR 'meningitis, recurrent' OR 'perimeningeal infections' OR 'recurrent meningitis' OR 'meningitides' OR 'pachymeningitis' OR 'pachymeningitides' OR 'coxsackie viruses' OR 'enterovirus' OR 'coxsackievirus' OR 'coxsackie' OR 'entero virus' OR 'coxsackieviruses’)

AND

('xpert ev' OR 'xpert enterovirus’)

**b. PubMed**

(Xpert EV) OR (Xpert enterovirus)

AND

(((((((((((((((Meningitis) OR (leptospirotic meningitis)) OR (meningeal inflammation)) OR (meningitis, recurrent)) OR (perimeningeal infections)) OR (recurrent meningitis)) OR (Meningitides)) OR (Pachymeningitis)) OR (Pachymeningitides)) OR (Enterovirus)) OR (coxsackievirus)) OR (coxsackie)) OR (entero virus)) OR (Coxsackieviruses)) OR (Coxsackie Viruses)) OR (coxsackieviruses))

**c. Cochrane Library**

Xpert EV OR Xpert enterovirus Meningitis

AND

"leptospirotic meningitis" OR "meningeal inflammation" OR "meningitis, recurrent" OR "perimeningeal infections" OR "perimeningeal infections" OR "recurrent meningitis" OR Meningitides OR Enterovirus OR "Coxsackie virus" OR coxsackievirus OR coxsackieviruses OR "entero virus" OR enteroviruses OR Coxsackieviruses OR "Coxsackie Viruses"

**d. Web Of Science**

**meningitis*** (Topic) or **"leptospirotic meningitis"** (Topic) or **"meningeal inflammation"** (Topic) or **"meningitis, recurrent"**(Topic) or **"perimeningeal infections"**(Topic) or **"recurrent meningitis"** (Topic) or **Meningitides*** (Topic) or **Pachymeningitis*** (Topic) or **Pachymeningitides***(Topic) **or coxsackieviruses***(Topic)**or Enterovirus*** (Topic)**or coxsackievirus*** (Topic) **or coxsackie***(Topic)**or Coxsackieviruses***(Topic) **or "entero virus"**(Topic)

AND

**(TS=(Xpert EV)) OR TS=(Xpert enterovirus)**
