# Supplementary material for: Evaluation of GeneXpert EV assay for the rapid diagnosis of enteroviral meningitis: a systematic review and meta-analysis
Source: Ann Clin Microbiol Antimicrob. 2022 Jun 9;21:25. doi: 10.1186/s12941-022-00517-3 (PMC9185958; doi:10.1186/s12941-022-00517-3)
Supplement: Supplementary file 3 — Additional file 3: S3. Screening processes. [file 12941_2022_517_MOESM3_ESM.pdf]

PRISMA 2009 Flow Diagram

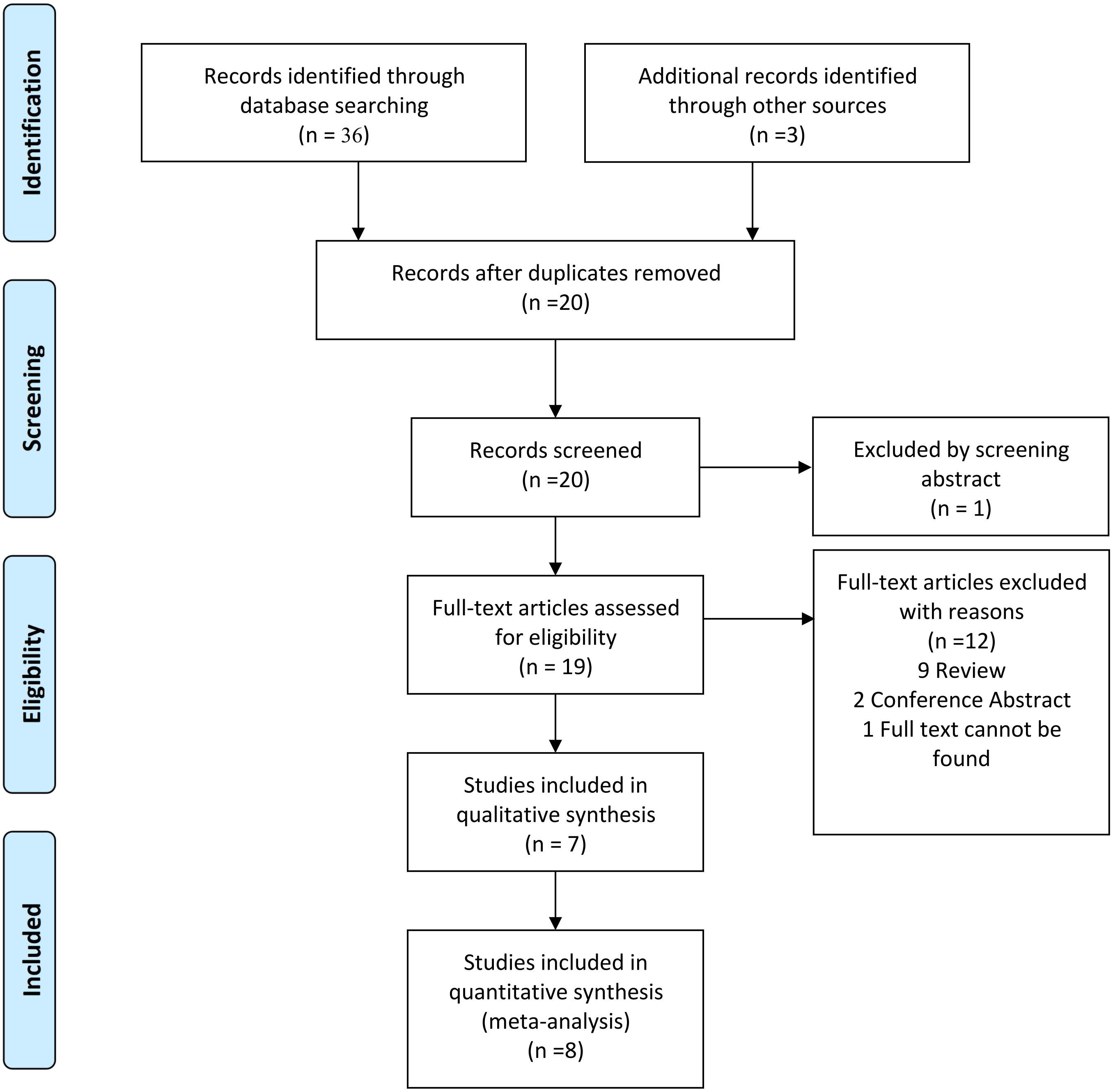


*From:* Moher D, Liberati A, Tetzlaff J, Altman DG, The PRISMA Group (2009). *P*referred *R*eporting *I*tems for *S*ystematic Reviews and *M*eta-*A*nalyses: The PRISMA Statement. PLoS Med 6(6): e1000097. doi:10.1371/journal.pmed1000097

**For more information, visit [www.prisma-statement.org](http://www.prisma-statement.org).**
